# Successful Bailout of Intraoperative External Iliac Artery Rupture by Aorto-Uni-Iliac Stent Grafting and Femoral-Femoral Crossover Bypass in a Patient With a Giant Common Iliac Artery Aneurysm

**DOI:** 10.7759/cureus.77349

**Published:** 2025-01-12

**Authors:** Hiroto Yasumura, Kenji Toyokawa, Keisuke Kawaida, Hideaki Kanda, Kosuke Mukaihara, Yoshiharu Soga

**Affiliations:** 1 Department of Cardiovascular Surgery, Graduate School of Medical and Dental Sciences, Kagoshima University, Kagoshima, JPN; 2 Department of Cardiovascular Surgery, Kagoshima City Hospital, Kagoshima, JPN

**Keywords:** access trouble, aorto-uni-iliac, bypass, ciaa, endovascular aortic repair (evar), excluder®, f-f, giant common iliac artery aneurysm, lunderquist®, rupture

## Abstract

Common iliac artery aneurysms (CIAAs) are typically asymptomatic and difficult to detect. As they enlarge and are identified at later stages, the risk of perioperative complications increases. Endovascular aortic repair (EVAR) is often a viable option for managing giant CIAAs. It is crucial to keep the tip of the guidewire in the descending aorta to ensure adequate torque transmission, to streamline the access pathway, and to address emergency situations such as aneurysm rupture or other access-related issues. Careful manipulation is essential, particularly in cases of severe tortuosity. However, complications may still occur. Here, we describe a fatal access-related complication involving the severance of the external iliac artery (EIA) and guidewire deviation into the retroperitoneal cavity during EVAR for a giant CIAA. This report is the first to document a bailout strategy for such an access-related complication in a patient with a giant CIAA. We present the case and our recovery approach with a literature review. An 88-year-old man presented with worsening left back pain. Contrast enhanced computed tomography (CT) revealed a giant left CIAA measuring 69 mm, with significant calcification extending from the abdominal aorta to both EIAs. EVAR was planned using a bifurcated Excluder® device. During the procedure, angiography of the left CIAA revealed a looped and tortuous EIA. While advancing a left DrySeal® sheath with a Lunderquist® stiff wire into the terminal aorta, the Lunderquist® slipped into the terminal aorta, causing the diameter of the DrySeal® loop to enlarge. As we attempted to retract the DrySeal® sheath to the distal EIA to avoid CIAA rupture, the loop enlarged further, and the patient suddenly went into shock. Assuming a CIAA rupture, we removed the stiff wire to the distal EIA to release the enlarged loop. However, angiography confirmed a rupture of the left EIA. Attempts to access the left EIA from the CIAA side using a through-and-through technique were unsuccessful. Consequently, we performed Excluder® aorto-right uni-iliac (AUI) stent grafting combined with a common femoral artery crossover prosthetic bypass using the upside-down technique. The patient’s postoperative course was uneventful, and he was transferred to a referral hospital on postoperative day (POD) 15. Unfortunately, three months after discharge, the patient succumbed to sepsis caused by methicillin-resistant *Staphylococcus aureus* (MRSA). In cases of severe tortuous access arteries that do not straighten despite the use of stiff wires, it is advisable to implement an early through-and-through technique between the brachial and femoral arteries. Additionally, proactive perioperative infection control, particularly for conditions like MRSA, is essential to improving postoperative survival and life expectancy.

## Introduction

Common iliac artery aneurysms (CIAAs) are rare, with an estimated prevalence of 0.02% in the general population [1]. These aneurysms are typically asymptomatic and challenging to detect, often only recognized when patients present with symptoms related to compression or rupture [2]. Several giant CIAAs (>10 cm) have been reported, including a 13 cm left-isolated arteriosclerotic CIAA [2], a 13 cm left-isolated inflammatory CIAA [3], and a 12 cm right-ruptured CIAA associated with a 17 cm aortoiliac aneurysm [4]. Consequently, CIAAs are frequently discovered at sizes well above the surgical threshold of >35 mm [5]. While rupture is rare for CIAAs <40 mm, aneurysms >50 mm are associated with a significantly elevated rupture risk [5].

Endovascular aortic repair (EVAR) is favored for giant CIAAs due to its less invasive nature. During EVAR, it is essential to maintain the guidewire tip within the descending aorta to ensure adequate torque transmission, to straighten the access route and aorta, and to facilitate rapid response to emergencies such as aneurysm rupture or other access complications. Careful manipulation is especially critical in cases with severe vascular tortuosity. Despite these precautions, complications can occur.

This report describes a case of fatal access-related complications during EVAR for a giant CIAA, involving the severance of the external iliac artery (EIA) and guidewire deviation into the retroperitoneal cavity. To our knowledge, this is the first report detailing a bailout strategy for such a complication in a patient with a giant CIAA. We describe the case and our recovery method with a literature review.

## Case presentation

An 88-year-old man (height: 148 cm, weight: 42.5 kg) presented with worsening left back pain over several days and difficulty walking. He arrived at the emergency department in a wheelchair. His medical history included tuberculosis in his 20s and unspecified rheumatoid polymyalgia, for which he was taking prednisone (2 mg/day). One year earlier, he had been diagnosed with a 65 mm left CIAA at another hospital but declined surgery, opting instead for antihypertensive therapy. Plain computed tomography (CT) at presentation revealed a giant left CIAA. The patient was prescribed an analgesic and agreed to undergo surgery.

He was subsequently referred to our hospital. On physical examination, the blood pressure was 136/75 mmHg, and the heart rate was 52 beats per minute. A pulsatile mass was palpable in the left lower abdomen and both posterior tibial arteries were palpable. Laboratory findings showed white blood cell (WBC) count of 7,580/μL, hemoglobin of 13.1 g/dL, platelet count of 131,000/μL, creatinine of 1.27 mg/dL, C-reactive protein (CRP) of 2.76 mg/dL, and fibrin/fibrinogen degradation products of 59.5 μg/dL. He had no pain on urination, but urinalysis showed WBC 2+ and urine culture was positive for methicillin-resistant Staphylococcus aureus (MRSA), which was not treated preoperatively. Electrocardiography revealed left axis deviation, complete right bundle branch block, and high left ventricular voltage. Transthoracic echocardiography revealed an ejection fraction of 57.5% without significant valvular heart disease. Coronary angiography indicated severe triple-vessel disease, which was managed perioperatively with aspirin, heparin, and vasodilators. Percutaneous coronary intervention with rotational atherectomy was planned post-EVAR.

**Figure 1:**
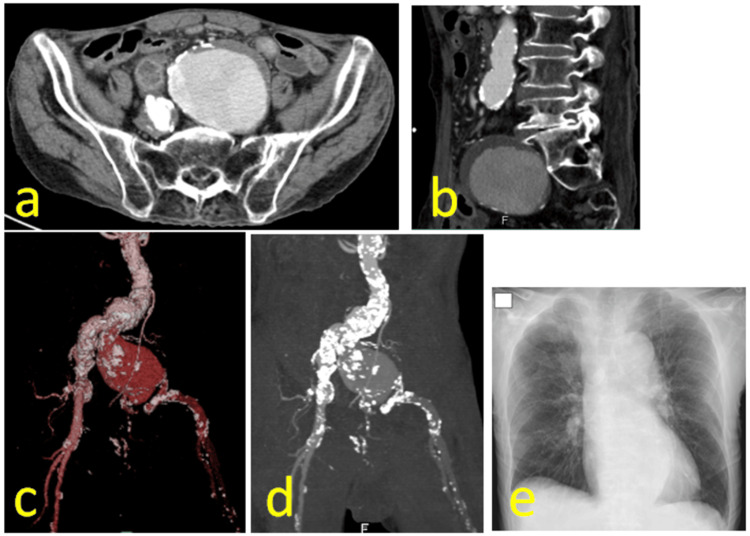
Preoperative findings (a) Contrast-enhanced CT showing a 69 mm left CIAA containing a mural thrombus. (b) The aneurysm was compressed by a sharp bone spur in the lumber vertebrae. (c) The abdominal aortic aneurysm measured 33 mm in diameter, and the right CIAA measured 29 mm. (d) Extensive calcification was observed from the abdominal aorta to both EIA. (e) Chest radiograph showing decreased transparency in the lung fields. CIAAs, common iliac artery aneurysms; EIA external iliac arteries; CT, computed tomography

Contrast-enhanced CT showed a 69 mm left CIAA containing a mural thrombus (Figure 1a). The aneurysm was compressed by a sharp bone spur in the lumbar vertebrae (Figure 1b). The abdominal artery aneurysm measured 33 mm, and the right CIAA measured 29 mm (Figure 1c). Extensive calcification was observed from the abdominal aorta to both EIAs (Figure 1d). Chest radiography revealed decreased lung field transparency (Figure 1e). Given the CIAA size, compression by a bone spur, patient age, severe triple-vessel disease, and extensive calcification, urgent bifurcated EVAR was planned.

To prepare for potential circulatory failure during surgery, bilateral femoral arteries and veins were exposed for percutaneous cardiopulmonary system placement. Prior to stentgrafting, occlusion of the left superior and inferior gluteal arteries was planned to prevent type II endoleak. After cannulation of 8 Fr short sheaths in both femoral arteries, a guidewire from the right femoral artery failed to cross the left proximal CIA stenosis due to calcification, and the occlusion was performed via the left femoral artery.

**Figure 2:**
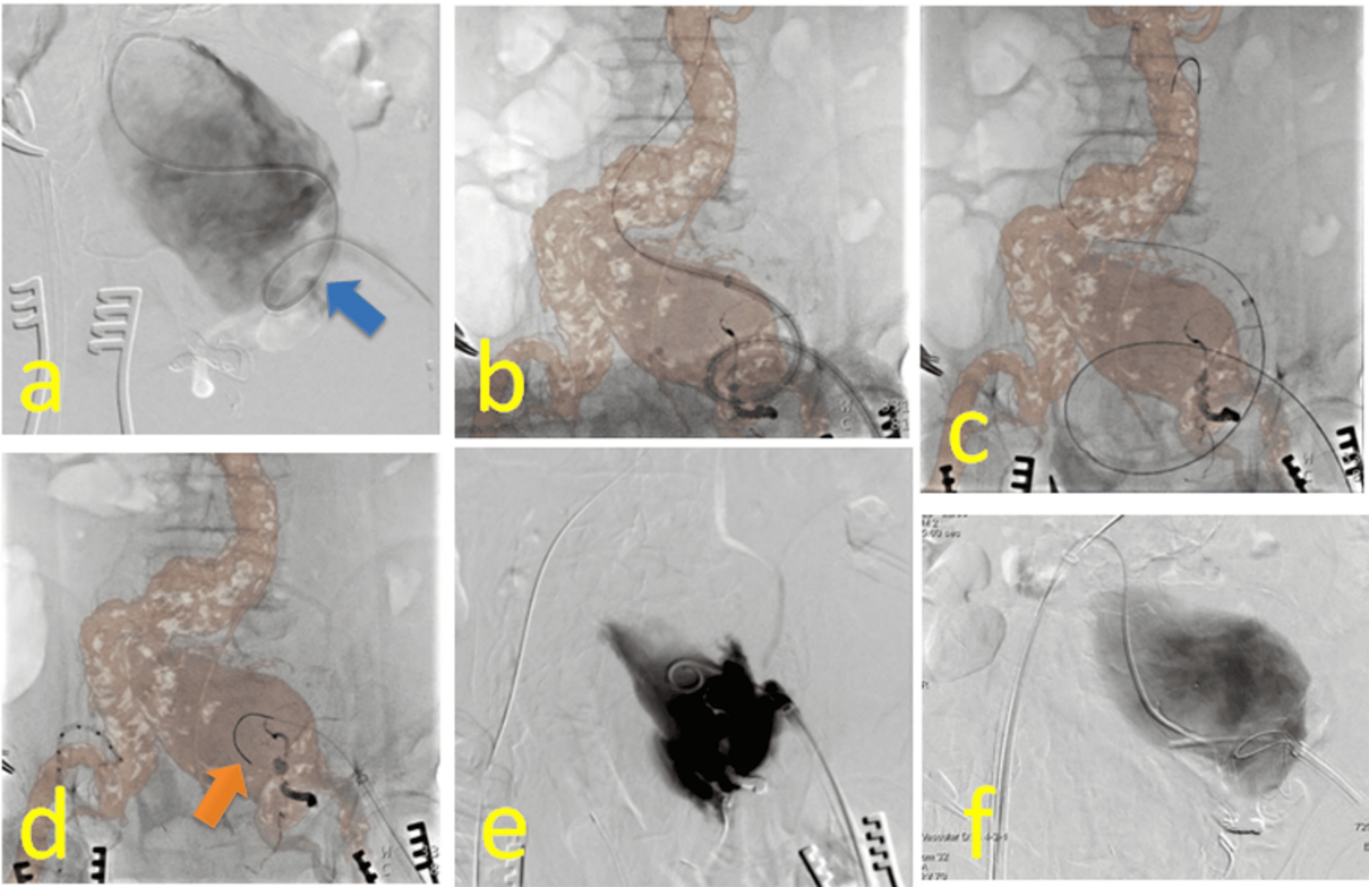
Intraoperative findings (a) Angiography of a giant CIAA showing a looped left EIA (blue arrow) and occlusion of the IIA. (b) The left tortuous iliac artery prevented the DrySeal® from advancing to the terminal aorta. (c) In order to avoid CIAA rupture, we began to withdraw the DrySeal® sheath, but the loop got more enlarged. (d) We assumed CIAA rupture and removed the stiff wire to the distal EIA in order to unleash the enlarged EIA loop. In reality, the tip of the Lunderquist® (orange arrow) was located in the retroperitoneal cavity. (e) Angiography of the left DrySeal® sheath showing extravasation into the retroperitoneal cavity. (f) A pigtail catheter with a guidewire was advanced via the right femoral artery to the left CIAA and tried to catch the guidewire via left femoral access with an Indy OTW® vascular retriever. CIAAs, common iliac artery aneurysms; EIA external iliac arteries; IIA, internal iliac artery

Considering C curve of the left iliac artery, we planned to deliver an bifurcated Excluder® via the left femoral artery. A pigtail catheter with a guidewire was advanced into the descending aorta via the left femoral artery. Angiography from the CIAA revealed looped and tortuous EIA (Figure 2a). The guidewire was exchanged for a stiff wire, Egoist®, to linearize the left iliac artery, and the 8 Fr sheath was upsized for 14Fr DrySeal® (Gore, New Jersey, USA) sheath in order to dilate and to linearize the access. However, the left tortuous iliac artery prevented the DrySeal® from advancing to the terminal aorta (Figure 2b). Therefore, Egoist® was switched to a stiffer wire, Lunderquist ® (COOK Medical, Indiana, USA). The tip of the DrySeal® was advanced to the terminal aorta, but the tip of Lunderquist® slipped down to the terminal aorta. The diameter of the DrySeal® loop was enlarging during the manipulation. In order to avoid CIAA rupture, we began to withdraw the DrySeal® sheath, but the loop got more enlarged (Figure 2c) and then systolic blood pressure dropped to 45 mmHg.

We assumed CIAA rupture and removed the stiff wire to the distal EIA in order to unleash the enlarged EIA loop (Figure 2d). Angiography from the left DrySeal® sheath showed extravasation from the left EIA to the retroperitoneal cavity (Figure 2e); thus, we suspected EIA rupture. We assumed that the EIA was only partially injured and planned a through-and-through technique. A pigtail catheter with a guidewire was advanced via the right femoral artery to the left CIAA and tried to catch the guidewire via left femoral access with an Indy OTW® vascular retriever (Cook Medical, Indiana, USA) (Figure 2f); however, it was impossible to catch the guidewire in the CIAA. We introduced 6Fr sheath in the left brachial artery and advanced a pigtail catheter to abdominal aorta for angiography and as another access.

**Figure 3:**
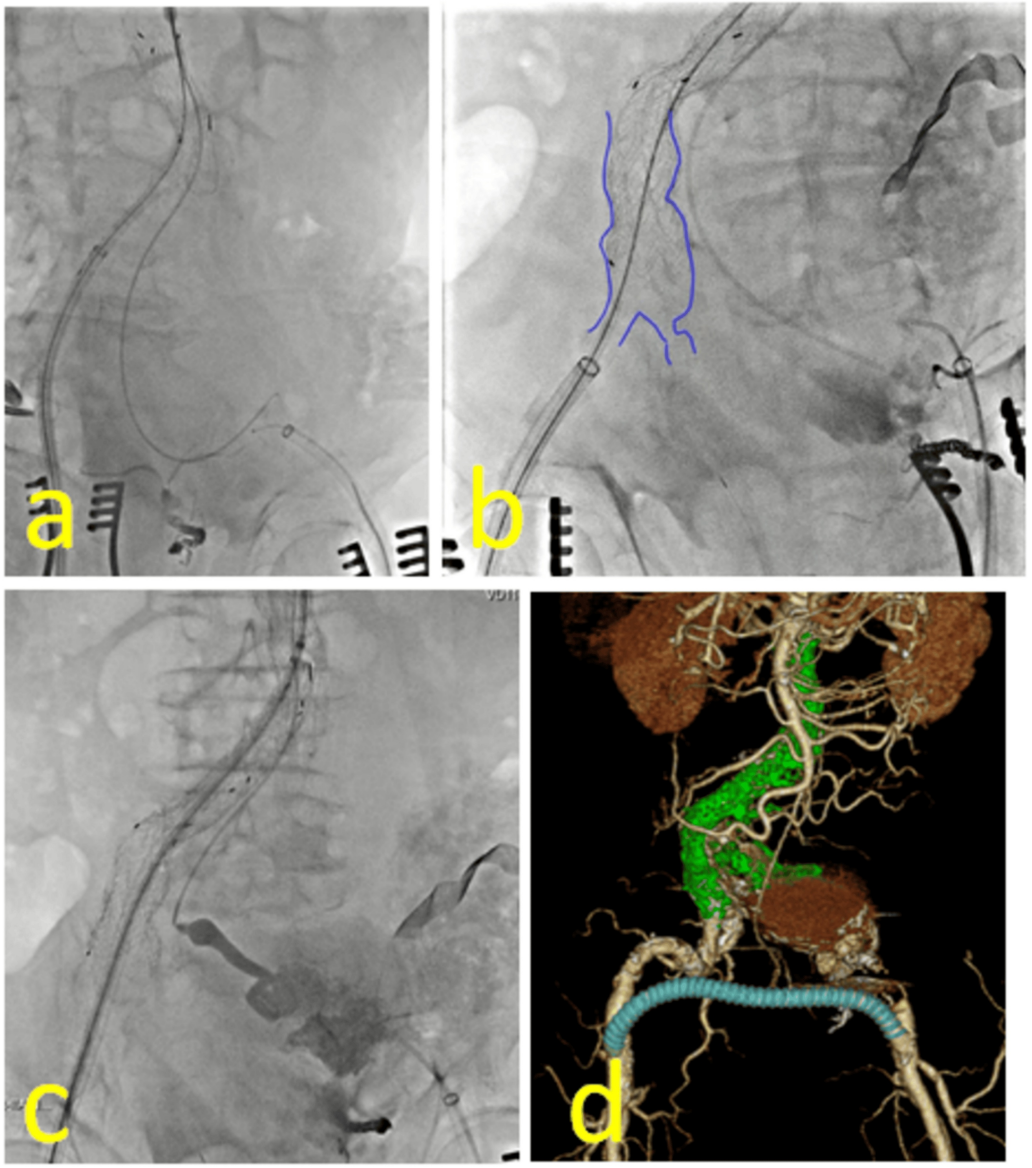
Intraoperative and postoperative findings (a) We advanced a pigtail catheter with a guidewire via the left brachial access to the left CIAA and attempted to catch the guidewire again in the CIAA via the left femoral access and to make a through-through, only to fail. (b) We delivered the upside-down stent graft via the right access using upside-down DrySeal® dilator whose distal edge was cut and deployed it in the trunk of the bifurcated Excluder® to occlude the contralateral opening. (c) The CIAA was embolized using n-butyl-2-cyanoacrylate via the left brachial access to control retroperitoneal bleeding. (d) Contrast-enhanced CT on POD 8 revealed a patency crossover bypass and no endoleak. CIAAs, common iliac artery aneurysms; CT, computed tomography; POD, postoperative day

A bifurcated stentgraft, Excluder® RLT231412J (Gore, New Jersey, USA), was deployed via the right femoral access, just below the renal arteries as planned. We advanced a pigtail catheter with a guidewire via the left brachial access to the left CIAA and attempted to catch the guidewire again in the CIAA via the left femoral access and to make a through-through (Figure 3a), only to fail. We realized that the left EIA was completely severed and that the edge of severed EIA was so spastic that the guide wire could not reach to Indy OTW®. Additionally we tried to expose the left EIA directly but mistakenly opened the abdomen. Massive bloody ascites prevented us from finding the left EIA. Therefore, we gave up a through-and-through technique,and decided to perform an aorto-right uni-iliac (AUI) grafting with common femoral artery crossover prosthetic bypass.

We prepared an Excluder® contralateral leg PLC231200J (Gore, New Jersey, USA), cut off the olive tip at the end of the stent graft and the deployment knob, extracted a stent graft from the device shaft, and refilled it upside-down into the device shaft. We delivered the upside-down stent graft via the right access using upside-down DrySeal® dilator whose distal edge was cut and deployed it in the trunk of the bifurcated Excluder® to occlude the contralateral opening (Figure 3b). Additionally, the CIAA was embolized using n-butyl-2-cyanoacrylate via the left brachial access to control retroperitoneal bleeding (Figure 3c). After touching up the stent graft with a balloon catheter, a femoral-femoral crossover bypass was constructed with Fusion® 8 mm graft (Getinge, Gothenburg, Sweden). The severed EIA was identified during abdominal exploration and we ligated the proximal and distal edge of the EIA with felted sutures.

The patient’s postoperative course was uneventful; he had no abdominal compartment syndrome and was extubated on the first postoperative day (POD). He was discharged from intensive care unit on POD4. Contrast-enhanced CT on POD 8 revealed a patency crossover bypass and no endoleak (Figure 3d). He was mobilized in a wheelchair and reported no back pain, with CRP of 2.54 mg/dL. On POD 15, the patient was transferred to a referral hospital. However, three months after discharge, the patient succumbed to MRSA-induced sepsis secondary to a urinary tract infection.

## Discussion

Both open surgical grafting and EVAR are viable options for managing CIAAs. However, in cases of giant CIAAs, open surgery carries high morbidity and mortality rates [6], with a significant risk of injury to pelvic veins, nerves, and ureters [5]. Additionally, the EIA and IIA are often located far distal to the aneurysm, making distal anastomosis technically demanding. As a result, distal anastomosis to the EIA may be abandoned, and the IIA is sometimes sacrificed in favor of anastomosis to the femoral artery only.

EVAR, though less invasive, also carries increased morbidity and mortality risks when calcified and tortuous arteries are used as access routes, due to the potential for occlusion or rupture. In the presented case of a giant CIAA, Y-grafting was considered but deemed unfeasible due to severe, near-circumferential calcification of the abdominal aorta. The anticipated challenges in achieving hemostasis and the patient’s poor cardiac and pulmonary reserves made EVAR the preferred option.

EVAR for giant CIAAs with severely calcified access pathways is rare and requires meticulous planning. The conventional approach involves IIA coiling and bifurcated stent grafting, which typically includes three or four components: the main body, ipsilateral leg, contralateral leg, and, if necessary, an iliac extender. Ensuring a linearized access route and abdominal aorta is critical to delivering the stent graft effectively. In our case, neither the Egoist® nor Lunderquist® stiff wires successfully linearized the left tortuous EIA, likely because their tips slipped down to the lower abdominal aorta, failing to transmit sufficient torque to the iliac artery. An early adoption of the through-and-through technique using stiff wires between the brachial and femoral arteries might have rectified this issue.

Monoiliac stent grafting combined with IIA coiling is another viable strategy for managing giant CIAAs. This approach involves extending the stent graft from the terminal aorta to the EIA. Bates et al. [2] used a low-profile aorto-uni-iliac converter (ZLC 24-66; Cook Aortic Interventions, Bloomington, USA), while Ikegaya et al. [7] employed an upside-down large contralateral leg PXC201000 (Gore, New Jersey, USA). Kaneda [8] reported success with a common femoral artery crossover prosthetic bypass followed by CIAA embolization. In our case, preoperative planning did not include a combination of IIA coiling and monoiliac stent grafting, which could have simplified the procedure given the adequate CIAA neck length.

In challenging stent grafting cases, the fundamental principle that bilateral guidewires must remain in the artery until the end of the surgery is imperative. In the event of aneurysm rupture or access-related complications, bilateral guidewires enable stent graft repair with proximal balloon dilation to control hemorrhage. Deviation from this principle can lead to difficulties in endovascular recovery and may necessitate conversion to open surgery.

Reflecting on a recent case, when the DrySeal® sheath through Egoist® could not advance to the terminal aorta, employing the through-and-through technique using the brachial and femoral arteries might have prevented EIA rupture. This approach could have allowed for options such as planned bifurcated EVAR or mono-iliac EVAR. In challenging access cases, the "tug-of-wire" technique should be considered after completing the through-and-through technique. This method involves exerting continuous traction on both ends of the wire at the brachial and femoral access points to overcome potential tortuosity in the aorto-femoral route while advancing the catheter [9].

From our case, we learned that when the DrySeal® sheath is advanced through a looped stiff wire, significant torque is transmitted to the vascular wall, not only during advancement but also during removal involving slip down of the stiff wire. This can enlarge the access loop and result in access severance. Additionally, when access injury occurs or is suspected, angiography should be performed immediately to identify the exact point of bleeding before manipulation of the guide wire. This could have prevented mistaking an EIA rupture for a CIAA rupture, ensuring the stiff wire tip remained in the aorta. When the Lunderquist® wire appeared to be removed to the distal EIA (Figure 2d), the wire tip had actually deviated into the retroperitoneal cavity. Therefore, advancing the DrySeal® sheath should be avoided when the stiff wire cannot adequately linearize the access, and the through-and-through technique should be considered early.

When performing AUI grafting with Excluder®, configurations such as combining the Excluder® main body with cuffs within the main body or placing the Excluder® leg upside-down, as described here, can be employed. The AUI stent graft configuration eliminates the need to consider the contralateral leg, making deployment technically less demanding than standard bifurcated grafting [10]. This strategy is applicable not only in the access-related complications mentioned above but also in cases of anatomically challenging access caused by severe arteriosclerotic stenosis or total occlusion. Although combined femoral-femoral crossover bypass may limit contralateral limb flow and pose a risk of bacterial infection, the versatility and effectiveness of AUI stent grafting with crossover bypass outweigh these drawbacks.

In the upside-down technique, the Excluder® stent graft does not require extracorporeal pre-deployment. The process involves unsheathing the delivery system in its collapsed state and reloading the upside-down collapsed stent graft into the delivery system. Deployment of the upside-down Excluder® [11] stent graft is a feasible and controlled method that enables precise placement by retracting the sheath over the dilator. In contrast, Zenith® [12] and Endurant® [13] stent grafts require extracorporeal pre-deployment and reinsertion, necessitating additional caution to prevent device damage.

Herein, we describe the successful bailout of an intraoperative EIA rupture using AUI stent grafting and femoral-femoral crossover bypass. Unfortunately, the patient succumbed to MRSA-induced sepsis and stent graft infection. This may have been partly due to the prolonged surgical procedure or compression of the urinary tube by a retroperitoneal hematoma. Thus, perioperative urinary tract infections, even without symptoms, should be proactively treated with antibiotics.

## Conclusions

We successfully managed an intraoperative EIA severance without a transluminal guidewire using AUI stent grafting and a femoral-femoral crossover bypass. This approach effectively excluded the aneurysm and maintained vascular flow. In cases where the access artery is severely calcified and tortuous and fails to straighten after the advancement of a stiff wire, an early adoption of the through-and-through technique between the brachial and femoral arteries with meticulous manipulation is crucial. Additionally, proactive perioperative infection control, particularly for conditions like MRSA, is essential to improving postoperative survival and life expectancy.

## References

[1] Brunkwall J, Hauksson H, Bengtsson H, Bergqvist D, Takolander R, Bergentz SE (1989). Solitary aneurysms of the iliac arterial system: an estimate of their frequency of occurrence. J Vasc Surg.

[2] Morgan-Bates K, Dey R, Chaudhuri A (2019). Ultrasound assisted on-table management of type III endoleak at endovascular repair of isolated giant common iliac aneurysm. EJVES Short Rep.

[3] Fokou M, Teyang A, Fongang E, Kamga J, Binam F, Sandmann W (2011). Surgical repair of a giant isolated inflammatory aneurysm of the left common iliac artery. Ann Vasc Surg.

[4] Galanopoulos G, Papavassiliou V (2020). A case of giant aortoiliac aneurysm rupture when open repair seems a one-way street. Aorta (Stamford).

[5] Wanhainen A, Verzini F, Van Herzeele I (2019). Editor’s choice - European Society for Vascular Surgery (ESVs) 2019 Clinical Practice Guidelines on the management of abdominal aorto-iliac artery aneurysms. Eur J Vasc Endovasc Surg.

[6] Melas N, Saratzis A, Dixon H, Saratzis N, Lazaridis J, Perdikides T, Kiskinis D (2011). Isolated common iliac artery aneurysms: a revised classification to assist endovascular repair. J Endovasc Ther.

[7] Ikegaya Y, Ogino H (2013). A case of placement of the reversed gore excluder large Conralateral leg for isolated iliac artery aneurysm (Japanese). Jpn J Vasc Surg.

[8] Kaneda M, Matsumoto K, Watada S (2004). A case of a pancreatic pseudocyst complicated with Hemosuccus Pancreaticus and giant aneurysm of the left common iliac artery. J Abdom Emerg Med.

[9] Ishimaru S, Kawaguchi S, Koizumi N (1998). Preliminary report on prediction of spinal cord ischemia in endovascular stent graft repair of thoracic aortic aneurysm by retrievable stent graft. J Thorac Cardiovasc Surg.

[10] Elkassaby M, Alawy M, Ali MZ, Tawfick WA, Sultan S (2015). Aorto-uni-iliac stent grafts with and without crossover femorofemoral bypass for treatment of abdominal aortic aneurysms: a parallel observational comparative study. Int J Vasc Med.

[11] van der Steenhoven TJ, Heyligers JM, Tielliu IF, Zeebregts CJ (2011). The upside down Gore Excluder contralateral leg without extracorporeal predeployment for aortic or iliac aneurysm exclusion. J Vasc Surg.

[12] Hiramoto JS, Reilly LM, Schneider DB, Rapp JH, Chuter TA (2009). The upside-down zenith stent graft limb. Vascular.

[13] Koike Y, Nishimura J, Hase S, Yamasaki M (2014). The upside down Endurant iliac limb stent graft for treatment of a common iliac artery aneurysm. Vasc Endovascular Surg.

